# Genetic variation in the TNF/TRAF2/ASK1/p38 kinase signaling pathway as markers for postoperative pulmonary complications in lung cancer patients

**DOI:** 10.1038/srep12068

**Published:** 2015-07-13

**Authors:** Michelle A. T. Hildebrandt, Jack A. Roth, Ara A. Vaporciyan, Xia Pu, Yuanqing Ye, Arlene M. Correa, Jae Y. Kim, Stephen G. Swisher, Xifeng Wu

**Affiliations:** 1 Departments of Epidemiology, The University of Texas MD Anderson Cancer Center, Houston, TX.; 2 Departments of Thoracic & Cardiovascular Surgery, The University of Texas MD Anderson Cancer Center, Houston, TX; and Division of Thoracic Surgery; 3 City of Hope National Cancer Center, Durate, CA.

## Abstract

Post-operative pulmonary complications are the most common morbidity associated with lung resection in non-small cell lung cancer (NSCLC) patients. The TNF/TRAF2/ASK1/p38 kinase pathway is activated by stress stimuli and inflammatory signals. We hypothesized that genetic polymorphisms within this pathway may contribute to risk of complications. In this case-only study, we genotyped 173 germline genetic variants in a discovery population of 264 NSCLC patients who underwent a lobectomy followed by genotyping of the top variants in a replication population of 264 patients. Complications data was obtained from a prospective database at MD Anderson. MAP2K4:rs12452497 was significantly associated with a decreased risk in both phases, resulting in a 40% reduction in the pooled population (95% CI:0.43–0.83, P = 0.0018). In total, seven variants were significant for risk in the pooled analysis. Gene-based analysis supported the involvement of *TRAF2*, *MAP2K4*, and *MAP3K5* as mediating complications risk and a highly significant trend was identified between the number of risk genotypes and complications risk (P = 1.63 × 10^−8^). An inverse relationship was observed between association with clinical outcomes and complications for two variants. These results implicate the TNF/TRAF2/ASK1/p38 kinase pathway in modulating risk of pulmonary complications following lobectomy and may be useful biomarkers to identify patients at high risk.

Successful surgical resection of non-small cell lung cancer (NSCLC) can dramatically improve long-term prognosis, with a 5-year survival rate over 50% in early stage patients who undergo surgery^1^. However, pulmonary complications are the major adverse event following resection with morbidity rates ranging upwards of 15%^2^,^3^. They are also risk factors for increased mortality, poor outcomes, and cost-of-care^4^,^5^. With advances in CT-based lung screening approaches, the number of patients with early stage NSCLC who are candidates for surgery may increase, underscoring the need for approaches to better predict which patients are at risk for pulmonary complications to reduce these events.

Several risk factors have been implicated in risk of pulmonary complications, including race, age, pre-operative pulmonary function, cardiovascular disease, smoking status, timing of smoking cessation, and chronic obstructive pulmonary disease^6^,^7^,^8^. However, these factors alone or in combination do not have strong ability to identify patients who will develop complications. Often those with favorable clinical profiles experience an adverse pulmonary event following resection. Therefore, there is a need for the identification of additional biomarkers that can enhance risk prediction and prevent or mitigate pulmonary complications.

Germline genetic variants are attractive biomarkers for clinical outcomes because they are stable, minimally-invasive, and do not rely on tumor tissue for assessment of risk. In this study, we hypothesized that common, germline genetic variation within a key inflammatory and stress response signaling pathway, TNF/TRAF2/ASK1/p38 kinase, would mediate risk of pulmonary complications following resection for NSCLC. This signaling pathway responds to cellular stress and inflammatory signals and activates pro-apoptotic pathways and cytokine cascades. The signal is initiated through binding of either TNF (tumor necrosis factor) or FASL (Fas ligand) to their receptors on the cell surface resulting in the formation of a complex that includes several mediators such as TRADD (TNFRSF1A-associated via death domain), FADD (Fas-associated via death domain), DAXX (death-domain associated protein), and TRAF2 (TNF receptor-associated factor 2). Together, these activate ASK1/MAP2K5, which through MAP2K4 (also known as MKK4) activates the p38 MAP kinases – alpha, beta, gamma, and delta (encoded for by *MAPK14, MAPK11, MAPK12,* and *MAPK13,* respectively). This kinase cascade results in the downstream transcription of target genes.

This study takes advantage of an extensive, prospective database that recorded pulmonary complications and other clinical variables in NSCLC surgical patients. This database together with availability of biospecimens provides an opportunity to investigate the genetic basis for these adverse events as a step towards developing an approach to identify high risk individuals.

## Methods

### Patient populations

Patients included in this study underwent lobectomy at the University of Texas MD Anderson Cancer Center between 1994–2009 for their histologically confirmed NSCLC. Patients were randomly assigned to discovery and replication populations while matching for age, gender, smoking status, and year of surgery. Written informed consents were obtained from all study participants. The study was approved by the Institutional Review Board of MD Anderson. All methods and analyses were carried out in accordance with this approval.

### Data collection

The Department of Cardiovascular and Thoracic Surgery at MD Anderson maintains an extensive prospectively entered database of lung cancer surgical patients. This database includes variables such as smoking behavior and co-morbidities prior to surgery, lung function tests, surgical procedure (type of surgery, chest wall resection, estimated blood loss, and intra-operative transfusion), surgical outcomes (vital status, number of days requiring ventilation, and length of hospital stay), and pulmonary complications. For this analysis, a pulmonary complication was defined as any adverse pulmonary event, such as pneumonia, acute respiratory distress syndrome, prolonged air leak, and atelectasis requiring intervention. Additional clinical and follow-up information was abstracted from patient medical records. Epidemiologic risk factors and demographic information were collected through an in-person interview using a structured questionnaire. Following each interview a 40 ml blood sample was drawn for DNA extraction.

### Genotyping

DNA samples were extracted from blood samples using QIAamp DNA extraction kit (Valencia, CA) and stored at −80 °C until use. Genotyping of 173 genetic variants in the TNF/TRAF2/ ASK1/p38 kinase pathway was performed using a custom Illumina iSelect BeadChip (San Diego, CA). Tagging SNPs were selected for each candidate gene based on data from the CEU population genotyped as part of the HapMap Project using the NCBI B36 assembly and dbSNP b126. Tagging SNPs were identified using Tagger^9^ with an r^2^ threshold of 0.8 and minor allele frequency ≥0.05 based on a region including +/−10 kb surrounding each gene. BeadChips were processed according the Infinium II assay protocol (Illumina). Quality control measures were applied to exclude SNPs and samples with poor call rates: 1) SNPs must have genotyping data from more than 95% of all samples and 2) samples must have genotyping data for more than 95% of all SNPs.

### Statistical analysis

Comparisons of the discovery and replication populations were analyzed using Student’s t-test, Mann-Whitney test, or Fisher’s exact test, as appropriate. Missing data was grouped as a separate category for analysis. Model-based selection was performed to identify variables that may potentially confound the analyses and those variables were included in multivariable logistic regression. The variance inflation factor was calculated to determine the independence of the final variables included in the multivariable analysis. Odds ratios (ORs) and corresponding 95% confidence intervals (95% CI) were estimated for each SNP adjusting for age at surgery (continuous), gender, inter-operative transfusion (yes/no), % DLCO predicted (continuous), and chest wall resection (yes/no). Higher-order gene-gene interaction analysis used the classification and regression tree (CART) analysis module in HelixTree software (Golden Helix, Bozeman, MT). Cumulative analysis was performed by summing the number of identified risk genotypes from the pooled analysis. Burden analysis was performed based on number of individual complications recorded for each patient. For overall survival analysis of the SNPs shown to be associated with complications, each SNP was fitted to the Cox proportional hazard model and hazard ratios (HRs) and 95% CI calculated adjusting for complications, gender, age at surgery, smoking status, stage, and treatment regimens. Kaplan-Meier survival functions and log-rank tests were used to assess overall survival durations with regard to genotype. All statistical analyses were performed using STATA (College Station, TX). Gene-based analysis was performed utilizing the statistical tool VEGAS^10^, which calculates a permutation-based P-value for each gene.

## Results

### Discovery and replication populations

The patient characteristics for the patient populations matched by age, gender, smoking status, and year of surgery are shown in Table 1. A total of 528 patients were selected for analysis based on those patients that had post-operative pulmonary complications data available and were enrolled in the case-control study that included genotyping of inflammation related genes. The mean age at surgery for both groups was 65 years and just over half were male patients. Fewer than 21% of patients were never smokers. Approximately 35–40% of patients experienced a pulmonary complication.

### Effect of TNF/TRAF2/ASK1/p38 kinase SNPs on pulmonary complications risk

Model-based selection identified five variables as potential confounders that were subsequently adjusted for in the multivariable logistic regression: age at surgery, gender, inter-operative transfusion, % DLCO predicted, and chest wall resection. The variance inflation factor for these variables ranged from 1.06–1.32, indicating that they are independent factors. In this adjusted analysis, eleven SNPs were significantly associated with pulmonary complications in the discovery population following (P < 0.05; Table 2). The most significant variant was in *TRAF2* encoding TNF receptor-associated factor 2. Individuals with at least one variant allele of rs6560652 had a 4.65-fold increased risk of developing complications (95% CI:2.03–10.68, P = 2.9 × 10^−4^). Two loci were associated with a reduction in risk – MAP2K4:rs12452497 and MAP3K5:rs13195420. Under the additive model, rs12452497 resulted in a 43% reduction (95% CI:0.35–0.92, P = 0.023) and patients with one or two rs13195420 variants had a 48% reduction (95% CI:0.29–0.91).

To rule out the possibility of false positive findings, the effects of the significant SNPs from the discovery population were assessed in a replication population (Table 2). MAP2K4:rs12452497 was associated with a 37% decrease in risk of pulmonary complication (95% CI:0.41–0.98, P = 0.039) under the additive model of inheritance. Two other SNPs, both in *MAP3K5*, reached borderline significance (P < 0.1) in the replication population with similar effects as the discovery population: rs9389421 (P = 0.063) and rs13195420 (P = 0.055).

Pooled analysis identified a total of seven variants that had consistent effects in both populations with a significant pooled P-value. MAP2K4:rs12452497 replicated in the replication phase and reached a combined P-value of 0.0018 in the pooled analysis (OR:0.60, 95% CI:0.43–0.83). Two SNPs with borderline significance in the replication population, MAP3K5:rs9389421 and MAP3K5:rs13195420 were significant in the pooled population with ORs of 1.92 (95% CI:1.25–2.93) and 0.55 (95% CI:0.37–0.81), respectively. The most significant association in the pooled population was for TRAF2:rs6560652, which was the top variant identified in the discovery phase. Although not significant in the replication population, pooled analysis showed that patients with at least one rs6560652 variant allele were 2.25-times (95% CI:1.38–3.66, P = 0.0011) more likely to develop a pulmonary complication compared to those with the common genotype. Similarly, one of the other top SNPs in the discovery population, TNF:rs18006299, which was also found to have a potential gene-gene interaction with TRAF2:rs6560652 (see below), was not significant in the replication population, but became significant in the pooled population (OR:1.55, 95% CI:1.02–2.34).

### Cumulative effect analysis

To quantitate the risk for each individual based on the number of risk genotypes, we performed a cumulative analysis in the pooled population (Table 3). A highly significant dose-response trend (P = 1.63 × 10^−8^) was observed with an increase in risk of pulmonary complications in those who carried a larger number of risk genotypes. In the pooled population, the risk for individuals with 4 to 6 adverse genotypes rose to nearly 4-fold (HR:3.95, 95% CI:2.40–6.49, P = 6.10 × 10^−8^).

### Higher-order gene-gene interaction analysis

**Figure 1:**
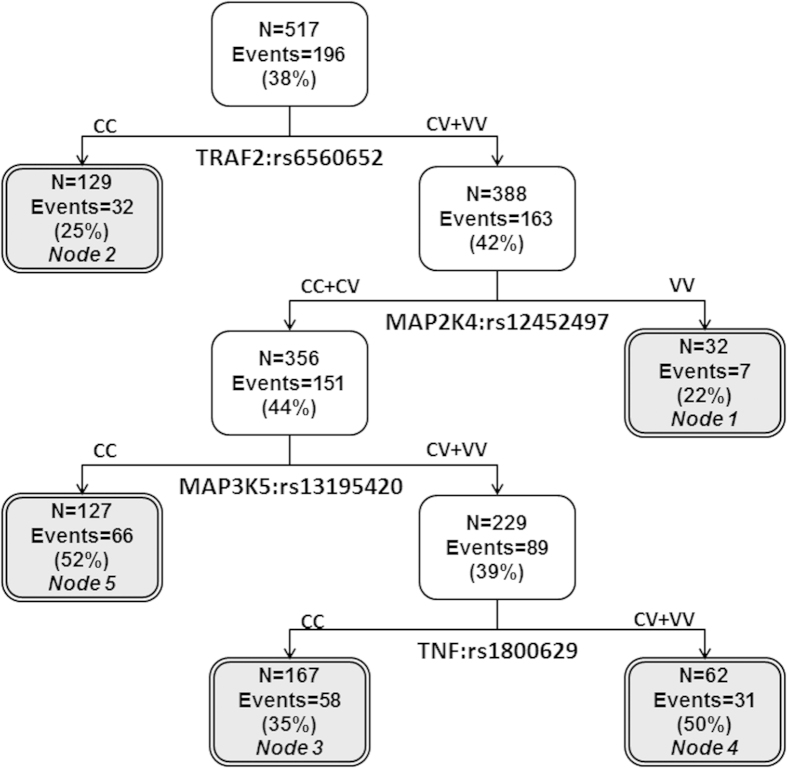
Potential higher-order gene-gene interactions among TNF/TRAF2/ASK1/p38 kinase signaling pathway variants. Abbreviations: C = common allele, V = variant allele.

We investigated whether there were interactions among the seven variants showing main effects in the pooled population that further modulated risk of complications. Indeed, potential higher-order interactions were observed with the initial split in the tree for TRAF2:rs6560652 with additional splits dictated by MAP2K4:rs12452497, MAP3K5:rs13195420, and TNF:rs1800629 (Fig. 1). The lowest risk nodes serving as the reference groups for the analysis were defined by the common genotype for TRAF2:rs6560652 (Node 2) and variant-containing genotypes for TRAF2:rs6560652 along with two variant alleles for the protective MAP2K4:rs12452497 variant (Node 1; Table 4). Individuals with the genetic background characterized by nodes 4 and 5 had the highest risk at nearly 4-fold (OR:3.95, 95% CI:2.34–6.34, P = 1.47 × 10^−7^), with over 50% of these individuals having a pulmonary complication. This increase in risk was much higher than that conferred by any individual SNP.

### Gene-based analysis

In addition to the effects of individual SNPs on risk of an adverse pulmonary complication, SNPs can combinatorially affect function of a gene. To provide evidence of the genes playing roles in pulmonary complications, we performed a gene-based analysis of risk. In this approach, the effects of SNPs within a gene are assessed while taking into account the linkage disequilibrium structure within that particular genomic region. The findings supported the CART analysis results and implicated *TRAF2* (gene-based P = 0.037), *MAP3K5* (gene-based P = 0.021), and *MAP2K4* (gene-based P = 0.020) as the top genes mediating pulmonary complications.

### Pulmonary complications burden analysis

Over a quarter of the patients (25.2%) in the pooled population had two or more pulmonary complications following surgery, with 7.8% experiencing three or more. Patients with a high complication burden would have a dramatically reduced quality of life following surgery that could also affect prognosis. MAP2K4:rs12452497, which was consistently associated with a protective effect, was also significant for protection against developing two or more pulmonary complications (OR:0.68, 95% CI:0.47–0.98, P = 0.037). This suggests that patients with the common genotype were more likely to have a high complication burden with multiple events following surgery. Similarly, individuals with two MAP3K5:rs13193586 variants were also associated with risk of two or more complications (OR:2.28, 95% CI:1.18–4.42, P = 0.014). Interestingly, DAXX:rs3130100, which was significant for complications risk in the discovery population but not in the replication or pooled populations, was significant for risk of more than two complications (OR:2.03, 95% CI:1.22–3.38, P = 0.006) and also more than three complications (OR:1.71, 95% CI:1.05–2.77, P = 0.031) compared to those with one or no complications under the dominant model.

### Relationship between risk of pulmonary outcomes and clinical outcomes

The occurrence of pulmonary complications has been previously found to be associated with a poorer outcome following resection of NSCLC tumors^4^. This was also found to be true for our population where patients with post-operative complications had a 1.43-fold (95% CI:1.02–2.00, P = 0.038) and 1.30-fold (95% CI:1.03–1.89, P = 0.03) increase risk of dying and progression, respectively compared to patients without complications. However, the effect of genetic variation on this relationship has not been investigated. We determined the effect of the seven significant complications risk SNPs for association with overall survival and progression. MAP2K4:rs12452497, the variant significant for complications risk in the discovery, replication, and pooled populations, was not associated with either outcome. Interestingly, TNF:rs1800629 was associated with survival benefit (HR:0.58, 95% CI:0.41–0.81, P = 0.002), in contrast to the overall 1.55-fold increase in pulmonary complication risk. Similarly, MAP3K5:rs13193586 was associated with a decreased risk of progression (HR:0.40, 95% CI:0.20–0.83, P = 0.014) although being associated with an increase in complications.

## Discussion

Pulmonary complications are the most frequent morbidities associated with resection for NSCLC and are associated with a poor prognosis. A range of clinical attributes have been investigated as predictors of individual risk^2^,^3^,^7^,^11^,^12^. However, there is a need for biomarkers that can enhance prediction and identify those at high risk of post-operative pulmonary complications. In this study, we identified several genetic variants within the TNF/TRAF2/ASK1/p38 kinase pathway that mediated risk of developing these adverse events, which not only provide potential biomarkers for prediction, but also implicates this key inflammatory and stress signaling pathway as playing a role in the physiological response to lobectomy in lung cancer patients.

A variant, rs12452497, in *MAP2K4* (also known as *MKK4*) was consistently associated with an approximately 40% reduction in risk in all three populations. This reduction in risk was also observed for complications burden. *MAP2K4* is located on chromosome 17 and is known for its role in the transduction of stress stimuli and inflammatory signals to mediate cellular responses, which includes apoptosis, cell growth, and inflammation^13^. Lobectomy for lung cancer would result in a dramatic increase in stress and pro-inflammatory signals that activates MAP2K4 and signaling through the MAPK stress pathway. Genetic variation that reduces activity or response of this key component of the pathway would result in an attenuated response and decreased risk of a pulmonary complication. Interestingly, although decreasing risk of a complication, this variant was not associated with overall survival or progression in these patients. This suggests that the effect of *MAP2K4* variation is in the immediate post-operative state and not the long term effect of lung cancer.

rs12452497 is located in an intron of *MAP2K4* and is a tagging SNP selected based on linkage disequilibrium patterns instead of potential function. Assessment of the genomic region surrounding rs12452497 identified another variant, rs4792219, in high linkage disequilibrium (r^2^ = 0.91) based on the 1000 Genomes pilot data located in the 3’-UTR of *MAP2K4*. However, there are no obvious regulatory elements disrupted by rs4792219 which would suggest potential functional effects. The other variants in high linkage disequilibrium with rs12452497 were located in intronic regions and also did not suggest functionality. Further investigation of *MAP2K4* will be needed to elucidate the biological basis for the observed association.

Two other loci, rs9389421 and rs13195420, reached borderline significance in the replication analysis. Both are intronic SNPs in *MAP3K5* (or ASK1) and are not in high linkage disequilibrium with any putative functional variants based on 1000 Genomes data. ASK1 activity leads to apoptosis in response to stress and inflammatory stimuli^14^. Although both are intronic, the differential effects on risk of developing a pulmonary complication indicate that the variants, or an undiscovered linked variant, have an effect through different mechanisms to enhance or reduce ASK1 function.

Potential higher-order gene-gene interactions analysis identified TRAF2:rs6560652 as the initial split. The resulting tree structure is comprised of four loci. Node 1 is particularly interesting because it suggests that the protective effect of MAP2K4:rs12452497 is able to overcome the effect of the risk conferring TRAF2:rs6560652 variant alleles, providing support that we need to take a comprehensive approach to analysis of genetic variation that does not evaluate each variant in isolation. Additional splits were created by MAP3K5:rs13195420 and TNF:rs1800629, creating the two highest risk nodes when in the context of the variant TRAF2:rs6560652 and common genotypes for MAP2K4:rs12452497. A majority of the patients with this combination of genotypes had a pulmonary complication (51.3%).

This analysis focused on the p38 kinase signaling component of the much larger and complex TNF signaling pathway that includes several other sub-pathways downstream of the cascade initiated by TNF and FAS signaling. There is extensive cross-talk between the TRAFs, MAP3K, and MAP2Ks that activate p38 kinases with the NFkB and JNK pathways^15^. The findings in this study point towards investigation of these other pathways to provide additional information regarding the biological function of global TNF signaling in the development of pulmonary complications, as well as the identification of additional novel biomarkers for clinical applications. Although appropriate biospecimens were not available for the current study population, it would be of interest to assess protein levels of the implicated genes either circulating or within the lung tissue and their correlation with genetic variation.

The major strength and advantage of this study was the availability of a prospective database from a single institution that has systematically recorded pulmonary complications and other variables associated with lung cancer surgery for well over a decade. This extensive dataset allowed us to design a hypothesis-driven study with both a discovery and replication population to rule out possible false-positive findings. Moving forward, replication in an independent population would be valuable to determine the transferability of these findings into different surgical settings. In addition, future research focusing on the interactions between these genetic variants, complications, and long-term effects such as survival, progression, and recurrence would be of interest.

Only a few studies have investigated the impact of common, germline genetic variation on pulmonary complications following surgical resection in lung cancer patients^16^,^17^,^18^. This includes a recent report from our group that showed the addition of six variants from the VEGF signaling pathway to a risk prediction model enhanced the prediction power by 6% from an area under the curve of 0.66 based on clinical variables to 0.72 with the addition of the variants^16^. This shift provides evidence that genetic biomarkers could provide clinically useful information for identifying patients at increased risk of developing complications. The current study expands our understanding of the genetic basis for the development of these complications and implicates the TNF/TRAF2/ASK1/p38 kinase pathway as playing a role in mediating these events. Ultimately, together with other established risk factors, this data may help to better identify those at risk as a step towards reducing complications and mortality associated with these events.

## Additional Information

**How to cite this article**: Hildebrandt, M.A.T. *et al.* Genetic variation in the TNF/TRAF2/ASK1/p38 kinase signaling pathway as markers for postoperative pulmonary complications in lung cancer patients. *Sci. Rep.* **5**, 12068; doi: 10.1038/srep12068 (2015).

## Figures and Tables

**Table 1:** Host characteristics.

	Discovery Population, N (%)	Replication Population, N (%)
Total	264	264
Age at Surgery, mean (SD)	65.41 (10.01)	65.53 (9.93)
Gender		
Male	141(53.4)	139(52.7)
Female	123(46.6)	125(47.3)
Smoking Status		
Never Smoked	54(20.5)	47(17.8)
>5yr prior	79(29.9)	89(33.7)
>12 mo – ≤5yr prior	26(9.8)	29(11.0)
>1 mo – ≤12 mo prior	37(14.0)	33(12.5)
>14 days – ≤1 mo prior	34(12.9)	33(12.5)
0–14 days prior	31(11.7)	31(11.7)
Missing	3(1.1)	2(0.8)
Stage		
I	174(65.9)	169(64.0)
II	37(14.0)	31(11.7)
III	51(19.3)	56(21.2)
IV	2(0.8)	8(3.0)
DLCO% predicted, mean (SD)	80.12 (20.12)	79.68 (19.49)
Year of Surgery		
1994–1999	41(15.5)	35(13.3)
2000–2005	126(47.7)	132(50.0)
2006–2009	97(36.7)	97(36.7)
Chest Wall Resection		
Yes	12(4.5)	17(6.4)
No	252(95.5)	247(93.6)
Intra-operative Transfusion		
Yes	12(4.5)	11(4.2)
No	252(95.5)	253(95.8)
Pulmonary Complications		
Yes	92(35.4)	103(40.1)
No	168(64.6)	154(59.9)
Missing	4	7

*some of the percentages may not be equal to 100% due to rounding.

**Table 2:** Results from top genetic variants associated with post-operative pulmonary complications.

			Discovery Population				Replication Population		Pooled Population	
Gene	SNP	bModel	OR (unadjusted)	P-value	aOR	P-value	aOR	P-value	aOR	P-value
TRAF2	rs6560652	dom	4.10 (1.97–8.51)	0.00015	4.65 (2.03–10.68)	0.00029	1.34 (0.71–2.53)	0.37	2.25 (1.38–3.66)	0.0011
MAP2K4	rs12452497	add	0.64 (0.41–1.00)	0.050	0.57 (0.35–0.92	0.023	0.63 (0.41–0.98)	**0.039**	0.60 (0.43–0.83)	0.0018
MAP3K5	rs9389421	dom	1.70 (0.98–2.94)	0.058	2.00 (1.08–3.72)	0.028	1.76 (0.97–3.19)	0.063	1.92 (1.25–2.93)	0.0027
MAP3K5	rs13195420	dom	0.60 (0.35–1.00)	0.051	0.52 (0.29–0.91)	0.023	0.58 (0.33–1.01)	0.055	0.55 (0.37–0.81)	0.0029
MAP3K5	rs13193586	dom	1.73 (0.82–3.65)	0.15	2.49 (1.08–5.75)	0.033	2.04 (0.78–5.30)	0.14	2.24 (1.20–4.18)	0.011
MAP3K5	rs13203080	rec	3.90 (1.14–13.34)	0.030	4.86 (1.14–20.74)	0.033	n/a	n/a	5.38 (1.33–21.72)	0.018
TNF	rs1800629	dom	2.10 (1.18–3.44)	0.011	2.46 (1.35–4.46)	0.0032	1.00 (0.55–1.82)	0.99	1.55 (1.02–2.34)	0.039
DAXX	rs3130100	dom	1.34 (0.77–2.35)	0.31	1.90 (1.01–3.57)	0.047	1.11 (0.61–2.02)	0.74	1.42 (0.93–2.18)	0.11
MAP2K4	rs7207011	dom	2.93 (1.01–8.50)	0.048	3.63 (1.11–11.82)	0.032	0.95 (0.35–2.54)	0.91	1.70 (0.82–3.50)	0.15
TNFRSF1B	rs17037696	dom	1.67 (1.00–2.79)	0.052	1.76 (1.01–3.08)	0.048	0.77 (0.44–1.32)	0.34	1.12 (0.76–1.65)	0.56
TRAF2	rs7019752	dom	2.06 (1.11–3.80)	0.021	2.17 (1.09–4.33)	0.028	0.55 (0.28–1.07)	0.076	1.04 (0.66–1.65)	0.86

^a^Adjusted for age at surgery, gender, intra-operative transfusion, % DLCO predicted, and chest wall resection.

^b^Model of inheritance: dom – dominant, rec – recessive, add – additive.

Bold indicates significance (P < 0.05) and underline indicates borderline significance (P < 0.10) in replication or pooled population.

**Table 3:** Cumulative effect of TNF/TRAF2/ASK1/p38 kinase signaling pathway variants on lung complications risk.

# of Risk Genotypes	Event, N(%)	No Event, N(%)	aOR(95%CI)	P-value
0 to 2	41(23.84%)	131(76.16%)	1(reference)	
3	56(38.89%)	88(61.11%)	2.10(1.23–3.59)	6.46 × 10^−8^
4 to 5	97(48.50%)	103(51.50%)	3.95(2.40–6.50)	6.10 × 10^−8^
P for trend	1.63 × 10^−8^			

^a^Adjusted for age at surgery, gender, intra-operative transfusion, % DLCO predicted, and chest wall resection.

**Table 4:** Higher-order gene-gene interactions among TNF/TRAF2/ASK1/p38 kinase signaling pathway variants modulating lung complications risk.

Nodes	Event, N(%)	No Event, N(%)	aOR(95%CI)	P-value
1 and 2	39(24.22%)	122(75.78%)	1(reference)	0.066
3	59(35.33%)	108(64.67%)	1.63(0.97–2.75)	
4 and 5	97(51.32%)	92(48.68%)	3.88(2.34–6.43)	1.47 × 10^−7^
P for trend	7.63 × 10^−8^			

^a^Adjusted for age at surgery, gender, intra-operative transfusion, % DLCO predicted, and chest wall resection.
